# The Integration of Psychophysiological Interventions with Psychotherapy and Pediatrics

**DOI:** 10.1007/s10484-025-09695-0

**Published:** 2025-04-04

**Authors:** Ethan Benore

**Affiliations:** Cleveland Clinic, Cleveland, OH USA

**Keywords:** Biofeedback, Psychotherapy, Children

## Abstract

There are established evidence-based interventions for children with various medical and psychological conditions. In addition, there is evidence supporting biofeedback to treat some of these conditions. However, there remains a gap in the literature in addressing how the practicing clinical psychologist or therapist can apply principles of psychotherapy to enhance biofeedback, as well as how components of biofeedback can enhance the application of evidence-based psychotherapies for children. This article utilizes a case-based approach to highlight some notable pathways for appropriate integration between psychotherapy techniques and biofeedback. It concludes with a summary of the current gaps and opportunities for research to address, as well as opportunities for clinicians and researchers to collaborate to better understand the real-world applications of successful integration of biofeedback with psychotherapy when treating children.

Clinical child and pediatric psychologists often find themselves working with children with combined medical and psychological conditions. While there is established evidence for psychological interventions, e.g., www.effectivechildtherapy.org (Wichers et al., 2023; Wuthrich et al., 2023), as well as growing data to support biofeedback interventions (Darling et al., 2020), there remains an opportunity to combine biofeedback with psychotherapy for enhanced effects (Lehrer, 2024; Steffen et al., 2024). Additionally, while data suggests that many psychologists are actively purchasing and using biofeedback, there is notably less effort in biofeedback training and applied clinical research (Benore & Banez, 2013), creating a gap in the application of biofeedback with clinical care. As one example, many children present with a combination of symptoms that may exclude them from a structured RCT protocolized treatment or may limit the applicability of RCT findings to their case. It remains the job (and the art) of the clinician to modify and apply evidence-based interventions to benefit the current child in front of them.

As a predominant clinician, I often encounter situations in which I lean on psychophysiological principles and biofeedback to enhance psychotherapeutic activities with children. It has been decades since application of cognitive behavioral therapy was restricted to “Thoughts, Feelings, & Behaviors” in my treatment of children, opening to a more inclusive application of physiology and self-regulation (e.g., Benore et al., 2014). However, clinicians also struggle demonstrating the impact of innovative therapy skills due to methodological limitations in day-to-day practice. This article uses a modified case-study approach to demonstrate instances where a clinician may creatively integrate evidence-based cognitive and behavioral interventions with psychophysiology and self-regulation training. While there are notable limitations to this method from a research standpoint, I aim to highlight the potential for future study and empower other clinicians to collaborate for applied clinical research in case series, quasi-experimental designs, and future more sophisticated research with children.

## Biofeedback to Enhance Relaxation Training

*"I'm not doing that breathing stuff."*

*"That breathing stuff doesn't work."*

These are common exclamations from children presenting in clinic. Breathing exercises are often a component of psychological interventions for a wide variety of conditions including, but not limited to, anxiety, depression, somatic symptoms, sleep, and toileting (e.g., Alneyadi et al., 2021; Karkouli et al., 2024; Zisopoulou & Varvogli, 2023). There is evidence to suggest the amount of practice will influence clinical outcomes (Greenberg et al., 2018), therefore adherence to breathwork matters. However, in practice, and potentially due to past pressure from insurance companies to limit the number of sessions, breathwork training may be reduced to a 5–10-minute exercise imagining a balloon in the child’s belly or else a simple recommendation to use an app. There are arguably many more recordings, relaxation scripts, and apps for children’s relaxation than there are training programs or competency evaluations for the therapist or child. This is important, as insufficient training can be construed as uncomfortable, interfere with adherence to training, and affect clinical outcomes (Schleicher et al., 2024).

In our clinic, we often have to backtrack the negative experience of a child with previous training in breathwork, who now presents with frustration and low confidence in the intervention. For example, in our program treating pediatric chronic pain, we have integrated biofeedback training to enhance the existing relaxation skills taught during group psychotherapy (Fahrenkamp & Benore, 2019). Biofeedback allows us to utilize data to enhance awareness, reduce bias, and increase adherence to a new relaxation training program.

The training protocol begins with an assessment of both respiration and heart rate variability, both during a 5-minute baseline (no-demand) condition as well as a 3-minute condition with active implementation of previously learned relaxed breathing. This assessment is enhanced with a thorough education about the respiration process and autonomic nervous system to directly address the child’s frustration (and noncompliance) with regular practice in breathwork. Often, therapy involves validating the child’s distress to improve rapport, identifying and challenging unhelpful cognitions associated with therapy, breathing, and bodily sensations, and enhancing self-efficacy and adherence. This is furthered with subsequent biofeedback-enhanced training in a modified practice of regulated diaphragmatic breathing. We then provide 24- and 48-hour follow-up on the practice, noting their positive sensory experience and pairing this with social reinforcement to enhance ongoing practice.

**Fig. 1:**
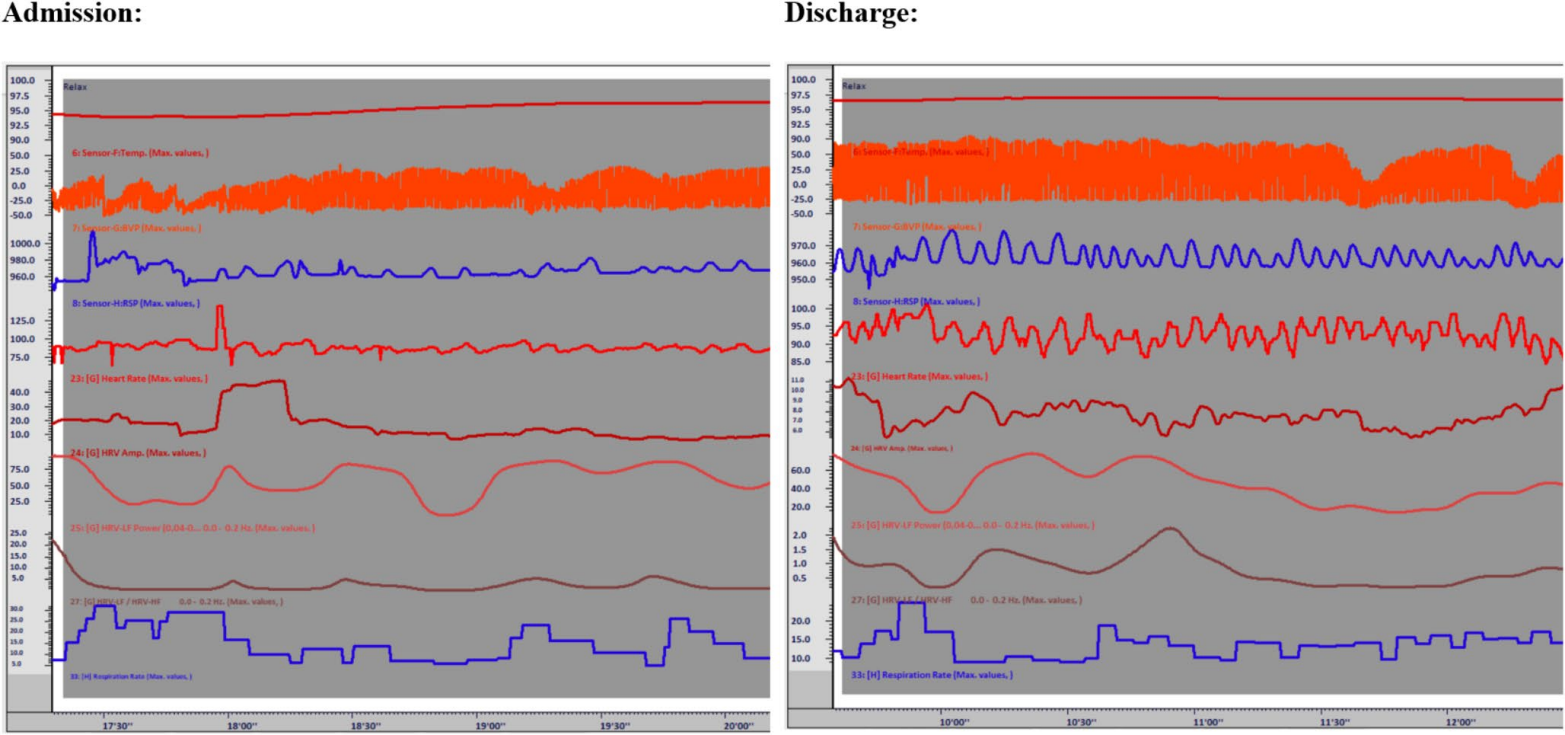
Screen shot of self-regulation at admission versus discharge for "Janice"

The most recent example was Janice, a 15-year-old white cis-gender female with Amplified Musculoskeletal Pain, Hypermobile Ehler’s Danlos Syndrome, and insomnia. She was admitted to our 3-week intensive interdisciplinary pain rehabilitation program for chronic pain. The weekly schedule within this program included 5 hours of individual occupational or physical therapy, 10 hours of group exercise programming (e.g., yoga, aquatics), 6 hours of recreational/art therapies, 6 hours of group and individual psychology, and 7 to 9 hours of school programming (Evans et al., 2016;see also Harrison et al., 2019). In addition to the above-mentioned therapies, Janice also received a protocolized heart rate variability (HRV) biofeedback intervention (utilizing Nexus-10 and BioTrace+ software), which allowed for standard assessment and training and with added unlimited practice with a personalized HVRB device (Inner Balance™). Data from key segments of biofeedback training are reported in Table 1; Fig. 1.

**Table 1:** Physiological data from biofeedback training for “Janice”

	RR	RR*	Temp	HR	HRV Amp	HRV LF	HRV LF%	LF/HF	SDNN	RSSMD
*Admission (no feedback)*										
Baseline (5 min)	14.60	NA	94.67	86.32	17.20	4229.2	68.47	2.91	83.50	71.53
Self-regulation (3 min)	14.90	10	95.26	88.76	15.28	1232.1	57.66	2.21	46.47	47.90
*Training session: Breathing*										
Self-guided Training (2 min)	10.40	9.5	70.06	85.90	12.85	1976.3	83.10	6.49	41.41	32.15
Pacer at 6.5 breaths/min (3 min)	9.07	7.5	70.16	83.81	15.34	3620.2	88.69	11.89	60.52	40.09
*Training session: Imagery*										
Baseline (2 min)	10.39	8.0	79.09	86.68	21.73	2724.3	78.43	5.53	69.18	46.29
Temp FB (Last 2 min of trial)	7.12	6.5	90.93	86.31	17.98	2198.0	86.02	8.37	67.70	32.05
*Training session: Increase HRV*										
Baseline (2.5 min)	18.89	8.0	71.02	88.32	36.13	5339.1	54.38	1.94	155.68	162.18
R+ LF% increase using puzzle	7.47	7.0	70.26	90.61	12.92	2640.5	87.22	15.28	42.01	22.84
*Discharge (no feedback)*										
Baseline (5 min)	14.28	16.0	96.88	92.94	9.65	624.7	47.04	1.11	30.23	18.17
Self-regulation (3 min)	13.69	15.0	96.84	92.46	7.68	249.8	43.19	2.23	22.84	15.62

*Note*: Additional biofeedback training occurred outside of data segments reported above. Also:

RR: Respiration Rate calculated from strain gauge belt placed approximately 1 inch above navel

RR*: Respiration Rate coded by visual inspection of trace line. Noted that thoracic breathing sometimes influenced calculated RR

HR: Heart Rate calculated from photoplesthysmograph (blood volume pulse) on non-dominant 2^nd^ digit

Artifact Rejection: Data was removed if excessive movement was detected (sEMG ampl > 100uV) or if heart rate exceeded the expected range (HR > 150)

**Fig. 2:**
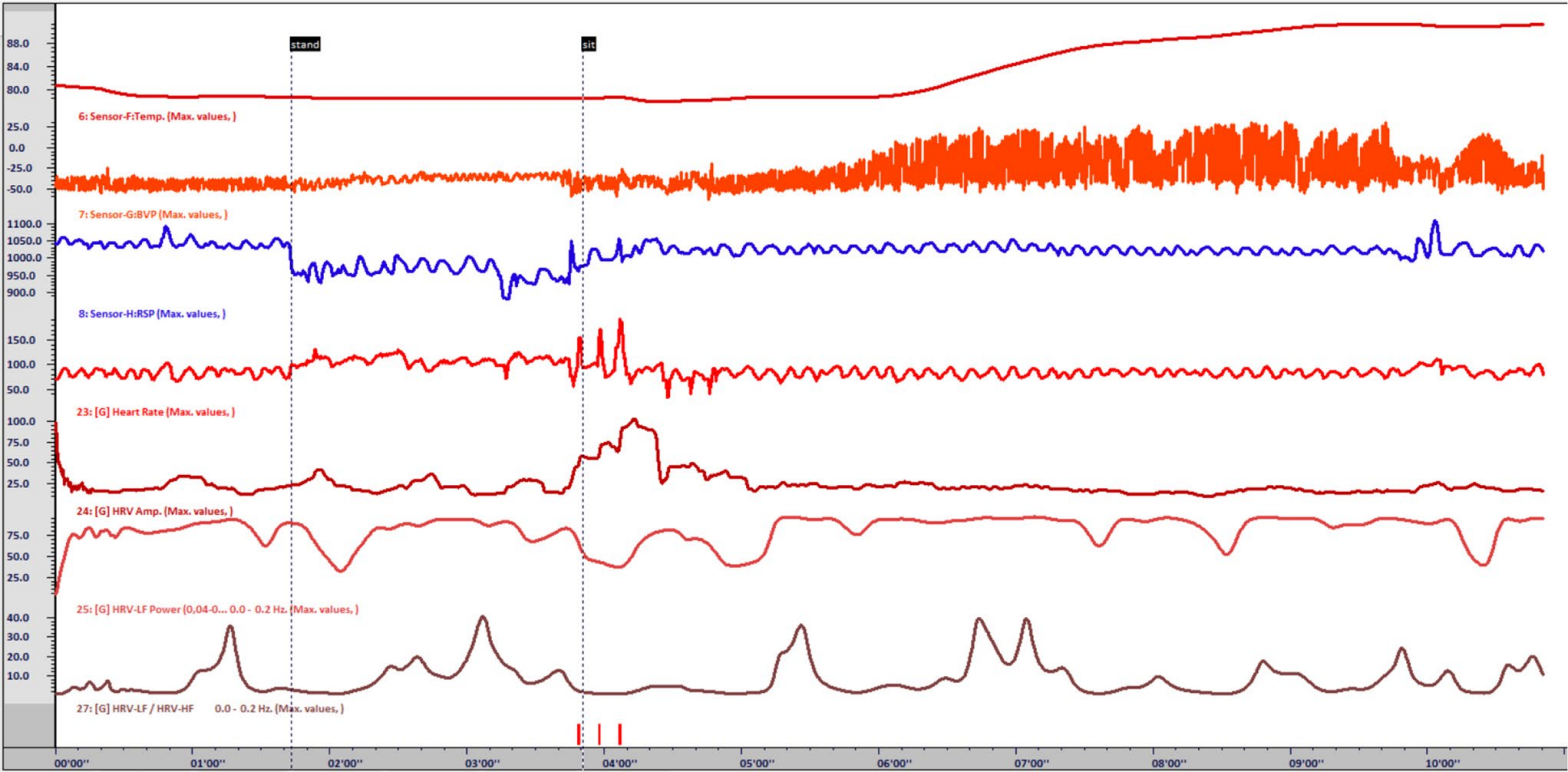
Screen shot of temperature biofeedback with self-guided imagery for “Janice”. Note: At 1’42”, asked Janice to stand. This demonstrated that HR altered but not to an extreme amount (as she feared) and corrected after sitting down at 3’51”. Note: by 5’00” Janice was actively implementing self-guided imagery paired with slow regulated breathing and witnessed the correlated changes in BVP and peripheral temperature, as well as HRV metrics

From a psychological perspective, Janice struggled with catastrophic thinking patterns, negative self-talk, and conflictual patterns of relating with others, which interfered with chronic pain rehabilitation. She had a history of single session training in “belly breathing” and reported training in mindfulness but could not detail what that training was. After biofeedback-assisted respiration training and a separate group-based training on self-guided imagery, Janice was able to experiment with slow regulated breathing paired with a pleasurable image spending time at the family farm (Fig. 2). We were able to link this with acute changes in physiology (using biofeedback), pleasurable mood states, and sustained adaptive physical functioning. This countered her past negative experience with relaxation training and readily encouraged her to modify her practice. While Janice did not like the handheld HRV biofeedback unit provided by the clinic, she then became self-motivated for personalized practice, continued it daily throughout the three-week program, and demonstrated independent ability to create and sustain adaptive physiological state measured through biofeedback prior to discharge (Fig. 1).

### Cognitive Restructuring to Enhance Temperature Biofeedback

A second effort involves pairing biofeedback with cognitive behavior therapy to enhance the biofeedback intervention. While one may describe biofeedback as operant conditioning, there is significant benefit in the therapist actively processing the data with the patient to enhance self-regulation.

In one example, biofeedback has proven helpful for Raynaud’s Syndrome (Daniels et al., 2018; Karavidas et al., 2006), although some would argue the evidence is mixed (Kondo et al., 2019). In clinical practice, taking time to understand the thoughts and emotions surrounding the condition may prove a gateway to self-regulation of symptoms.

Several years ago, Bobby, a 16-year-old white cis-gender male, presented with previously diagnosed Raynaud’s Syndrome. He presented with other psychological issues including intermittent drug use and mild conduct disorder, but most distressing for him was his “cold sweaty hands.” We were able to pull from Acceptance and Commitment Therapy (ACT, e.g., Binder et al., 2024; Petersen et al., 2024) to support treatment. For Bobby, this involved utilizing cognitive diffusion and a clarification of his values. Conversations while connected to biofeedback supported an increased awareness of autonomic functioning accompanying changes in cognitive states. Put simply, Bobby wanted to hold a girl’s hand but was often too embarrassed to even attempt physical touch. Using biofeedback (utilizing Nexus-10 and BioTrace + software), Bobby was able to readily create a calm state, but when paired with imagined exposure, he demonstrated changes in both EDA and peripheral temperature (i.e., cold and sweaty hands). While we utilized these as endpoints for training, we agreed to add photoplethysmography to allow for a quicker response time in terms of increasing blood volume in his fingers. Paired with a modified assessment, Bobby was able to challenge automatic negative self-talk, maintain optimism for his goals and values, and see in real-time how this shift in mindset actually moved him closer to his goal. He was then able to demonstrate conscious control of the Raynaud’s phenomenon; unfortunately, he did not return for further training.

A second example involved Isabella, a 12-year-old cisgender female of Mediterranean descent presenting with problematic vocal tics. Habit-reversal training is an evidence-based treatment for tic disorder (Hollis et al., 2016; Shou et al., 2022). With vocal tics, at times a relaxed style of breathing is taught as the replacement behavior. Given patient interest in biofeedback to enhance self-efficacy in tic management, we utilized several modalities (respiration, HRV, temperature) to support training (utilizing a J&J Engineering 4-channel instrument). Breathwork and HRV training proved to increase her anxiety and frustration due to the rapid physiological response and amplified signals displayed to her—she was overly eager to perform well and therefore hypercritical of the physiological feedback. We opted to utilize temperature biofeedback to support operant training in a prolonged state of calm. Unfortunately, as she progressed in therapy and self-regulation, she because increasingly frustrated with tic presentation minutes into the session. Through a therapeutic review of biofeedback data and tic presentation, we identified that Isabella experienced automatic negative self-evaluations when even one tic presented after 1–2 minutes of a period of calm. Isabella worried that she did not have full control of her tics and never would, thereby increasing her tension, arousal, and tic presentation. Using ACT methods of cognitive diffusion and acceptance, Isabella was able to allow these thoughts to occur and put them in a larger value-based and goal-oriented context. Specifically, Isabella changed her mindset to: “*I am capable of relaxing. As I am settling down*,* 1 or 2 ticsmayemerge*,* but these are the last tics my body is letting go of as it fully settles.*” This mindset shift allowed Isabella to more readily engage in the exercise and feel confident and satisfied with her habit reversal training.

## Biofeedback to Enhance Exposure Therapy

One of the more powerful case examples of applications of combined heart rate variability biofeedback and psychotherapy was Allie, an 18-year-old white cisgender female with significant needle phobia. Her pediatrician referred her for support receiving required vaccinations as required by her college. An initial attempt at vaccination resulted in her collapsing to the floor in tears and curled in the fetal position with an extreme fear of needles. Evidence suggests that several strategies can support treating needle phobia in children and adolescents. A recent meta-analysis indicated varying level of support for distress and pain management with distraction, breathwork, CBT, and hypnosis, while there was no evidence supporting preparatory information, suggestions, or memory alteration alone (Birnie et al., 2018). However, we were able to pair distraction, breathwork, and CBT for a successful intervention.

Allie first utilized the Heartmath EmWave™ program to train self-regulation. This strategy was chosen as it presented a relatively easy method to support and monitor Allie’s efforts at counter-conditioning. The initial target was to target was to down-regulate anxiety with paced breathing and some added distraction. This technique of HRV biofeedback was then paired with exposure trials to a mock needle stick—this technique was chosen as we had the opportunity to replicate HRV biofeedback during the actual vaccination procedure.

The first exposure trial involved me playing the role of a mock nurse wearing gloves and gently holding Allie’s hand in position for her needle stick. The Subjective Units of Distress Scale (SUDS ratings) from 0 to 10 were used to monitor anxious arousal. As predicted, this exposure trial was enhanced for Allie through a distracting and engaging activity that directly reinforced a calm state—HRV biofeedback-assisted relaxation. After multiple trials with low levels of exposure, we increased the intensity of the mock needle procedure: first holding a blunted pen in hand as mock needle, pressing the pen to the skin, then holding an actual capped needle in hand, pressing the capped needle to skin to simulate a needle stick. Allie was able to successfully tolerate the mock procedure both with eyes open and closed after successive trials, with low SUDS ratings. Unfortunately, the initial date of actual vaccination was rescheduled. Importantly though, Allie chose to proceed on another day without therapeutic assistance, tolerated the vaccination and transitioned to college successfully.

## Biofeedback to Enhance Self-regulation in Autism

Children with autism often struggle with self-regulation. This may be related to sensory overload or poor somatosensory awareness (Coffman et al., 2024; Riquelme et al., 2023; Wodka et al., 2016) and difficulties turning abstract concepts such as emotional states and core beliefs into language that can be addressed in therapy (Dallman, 2024; Zinck et al., 2021). However, in teens with high-functioning autism, peripheral biofeedback may help to make the abstract inner world of the child more concrete, supporting education on emotions and relaxed states. Further, specific exercises can be paired with this information reinforced, similar to past attempts at behavioral relaxation training (Paclawskyj, 2011). When teenagers with high-functioning autism present to our pediatric chronic pain rehabilitation program, we have utilized biofeedback to simplify the process of self-regulation for them. This is especially helpful when attendance at our group-based mind-body skills training sessions does not transfer into positive change for the patient.

**Fig. 3:**
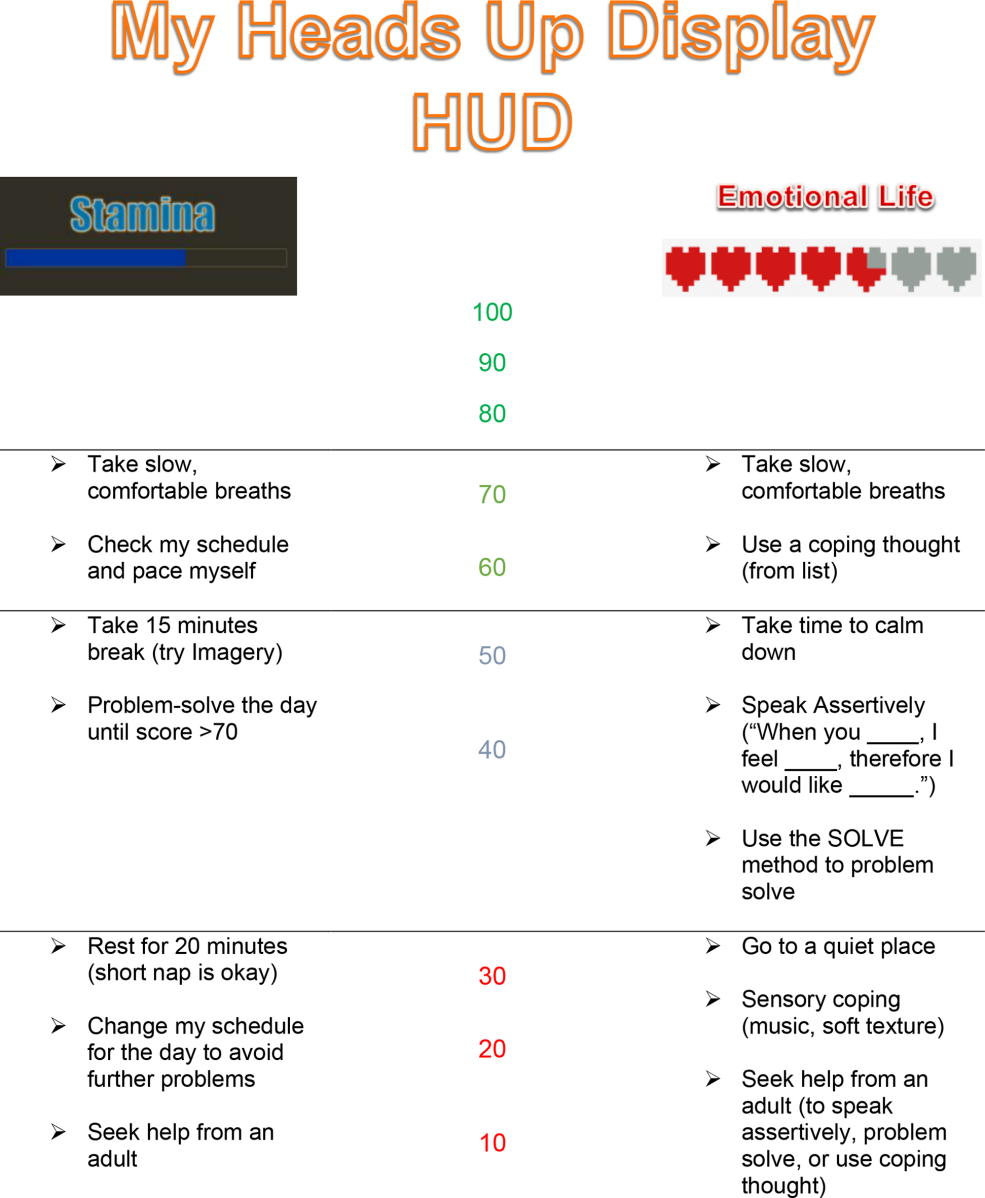
Heads-Up-Display for “Vanessa” to prompt and shape self-regulation

One example is Vanessa, a 14-year-old cis-gender white female with chronic pain and social anxiety, admitted to our pain rehabilitation program. She initially presented to my office with a hoodie zipped all the way up through the hood, covering her entire face. Face-to-face social interaction was distressing and difficult. Biofeedback allowed the initial therapy to take place with minimal direct face-to-face communication. She enjoyed video games and accepted that a therapist could assist her at performing better at a game-like intervention in biofeedback. After some gains were made in biofeedback assisted self-regulation, the transfer of skills into real-world application needed attention. Since Vanessa was fond of video games, she understood and appreciated the concept of an HUD (heads up display), where a video game character on the computer is actively monitored by ‘life points,’ ‘magic points,’ and the like. Using biofeedback as a launch pad to somatosensory awareness and communication, we were able to develop her own HUD focusing on Stamina (physical wellness or vitality) and Emotional Life (calm happy state), which she could visualize and communicate much more effectively (Fig. 3). We then turned this grading system into a 0 to100 scale, pairing each level with specific behavioral strategies that would help with self-regulation. For example, when Stamina dropped to 70, Vanessa’s HUD prompted her to take a brief break and ground herself, sitting still for 2–3 min and breathing slowly. Likewise, when Emotional Life dropped to 50, she was prompted to use a template to speak with assertiveness and/or use a problem-solving mnemonic. When either meter dropped below 30, she was prompted to reduce external stimulation using some grounding techniques and follow the direct advice of a trusted adult. And if both meters were above 80, no specific intervention was needed—Vanessa was successfully self-regulating!.

## Integrating Therapy and Biofeedback with Children: Current and Future State

As a psychologist working with children, I have long been fascinated by the concepts of self-regulation, particularly physiological self-regulation and biofeedback. Perhaps unfortunately, research on evidence-based psychotherapy for children either does not include biofeedback or does not accurately address *how* biofeedback or self-regulation strategies are effectively employed in treatments. Either biofeedback is evaluated by adherence to a specific protocol (e.g., Sowder et al., 2010) or referenced as part of a larger evidence-based interventions without a clear description of the application (Fisher et al., 2018). There remains significant work to be done in the research arena to enhance the integration of psychotherapy and biofeedback. For example, despite long time and widespread use of diaphragmatic breathing, one systematic review did not conclude it was even beneficial for children (Barker et al., 2013). A separate meta-analysis underscored the improved quality needed in research on pediatric biofeedback to truly validate its effectiveness (Darling et al., 2020).

This gap in the intersection between research and practice often proves challenging to the practicing clinician. Both applied clinical research studies and review studies have limitations, either through insufficient methods or data to support the validity of findings or over-specification of the clinical population that may limit the external validity of such techniques. Further complicating this are clinicians who are either not fully trained in biofeedback or not actively addressing the knowledge gap in application of biofeedback to the treatment of children (Benore & Banez, 2013). That said, there are a number of evidence-based therapies to treat children’s disorder that could be enhanced by biofeedback, and one fruitful approach may be to better study the integration of biofeedback into psychotherapy. Targets of study could be facilitation of emotional awareness, emotional expression, cognitive diffusion (e.g., separating physiological arousal from cognitions), graduated exposure, and concrete goal-directed behaviors. As in many fields of science, this will begin with less rigorous clinical observation such as the case examples above. Clinical observations will lead to greater case studies. This will inform both additional bench research on psychophysiology as well as the development of larger methodologically sound trials. Improved collaboration between academic researchers and full-time clinicians should enhance this endeavor.

Upon further reflection, it is also important to recognize the potential bias in biofeedback with children. In many cases, developers of technology focus on the “gamification” of biofeedback. While this clearly has a role, it should be underscored that in only one example above did a patient utilize ‘feedback’ beyond an analog bar graph or line graph. As a clinician the ‘art’ of therapy in this regard is translating the data of biofeedback into real world applications. Clinicians who overly focus on the biofeedback program, the mindfulness app, or the previously published self-regulation script are doing themselves and their patients a disservice. My own practice draws from my past training in biofeedback of somatosensory awareness, enhanced personal control of autonomic functioning, and generalization to meet personal goals including symptoms reduction. Psychotherapy skills can further support these techniques, but future literature must demonstrate how.

## Data Availability

Data provided is non-disclosed case example and cannot be shared without first seeking permission from the patient.
